# Supplementary material for: Platelet-Derived Extracellular Vesicles Stimulate Migration through Partial Remodelling of the Ca2+ Handling Machinery in MDA-MB-231 Breast Cancer Cells
Source: Cells. 2022 Oct 4;11(19):3120. doi: 10.3390/cells11193120 (PMC9564117; doi:10.3390/cells11193120)
Supplement: Supplementary file 1 [file cells-11-03120-s001.zip › cells-1940806-supplementary.pdf]

Supplementary Information

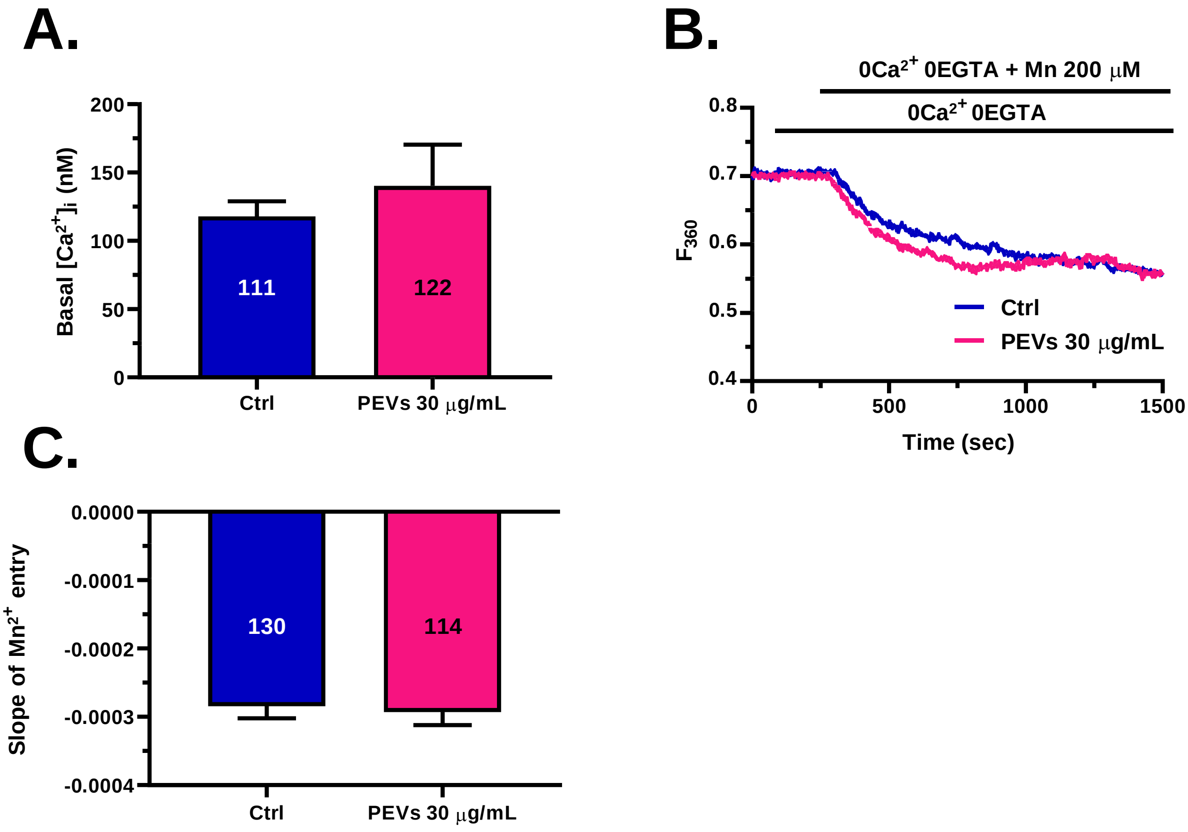

Figure 1. Long-term exposure of MDA-MB-231 cells with PEVs did not affect basal [Ca^{2+}]$_i$ and constitutive Ca^{2+} entry. A. Evaluation of resting [Ca^{2+}]$_i$ did not reveal any difference between control (Ctrl) cells and cells treated with PEVs (30 µg/mL, 24 h). Basal was evaluated by using the Grynkiewicz equation, as shown elsewhere [35]. **B.** Resting Ca^{2+} entry in MDA-MB-231 cells was evaluated by using the Mn^{2+}-quenching technique, as described in Materials and methods. The extracellular physiological solution was first replaced with a 0Ca^{2+} solution and then 200 µM Mn^{2+} was added to cause an immediate decay in Fura-2 fluorescence, which is consistent with the occurrence of constitutive Ca^{2+} entry. Resting Mn^{2+} influx in MDA-MB-231 cells was not impaired by the treatment with PEVs (30 µg/mL, 24 h). The rate of fluorescence decay for each individual tracing was calculated as the slope of a linear regression. **C.** Mean ± SEM of the quenching rate of Fura-2 fluorescence induced by Mn^{2+} addition in control (Ctrl) and treated cells.

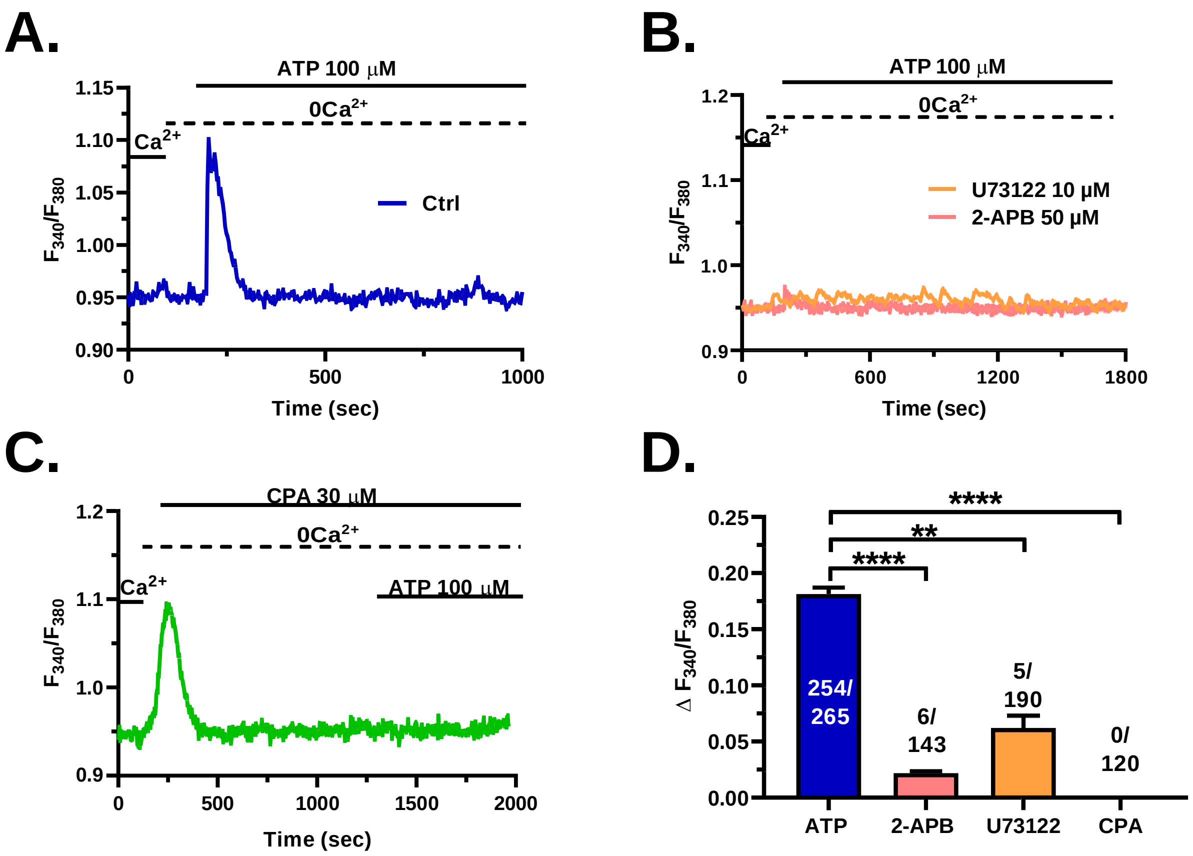

Figure S2. $InsP_3$-dependent ER Ca^{2+} mobilization supports ATP-induced intracellular Ca^{2+} release in MDA-MB-231 cells. A. Intracellular Ca^{2+} release evoked by ATP (100 µM) in untreated (Ctrl) MDA-MB-231 cells in the absence of extracellular Ca^{2+} ($0Ca^{2+}$). **B.** ATP-evoked intracellular Ca^{2+} release was largely suppressed by blocking PLCβ activity with U73122 (10 µM, 30 min) and inhibiting $InsP_3Rs$ with 2-APB (50 µM, 30 min). **C.** ATP-evoked intracellular Ca^{2+} release was abrogated upon depletion of the ER Ca^{2+} pool with CPA (30 µM) under $0Ca^{2+}$ conditions. **D.** Mean±SE of the amplitude of ATP-evoked endogenous Ca^{2+} release under the designated treatments. **** indicate $p < 0.0001$, ** indicate $p < 0.01$.

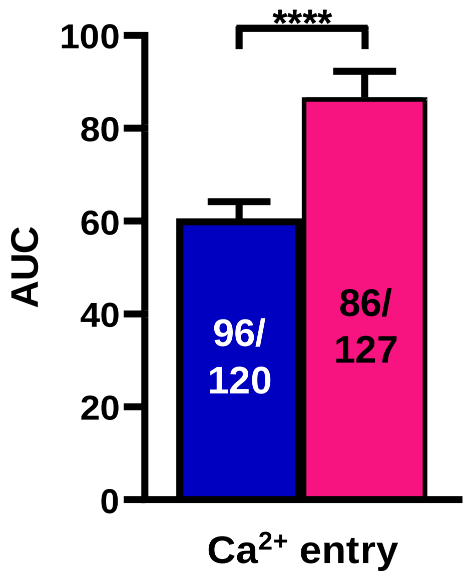

Figure S3. Further evidence that long-term exposure of MDA-MB-231 cells with PEVs increases FBS-induced extracellular Ca^{2+} entry. Measurement of the AUC (mean±SE) confirmed that pre-treatment of MDA-MB-231 cells with PEVs (30 μg/mL, 24 h) significantly increased 20% FBS-induced extracellular Ca^{2+} entry. **** indicate $p < 0.0001$.

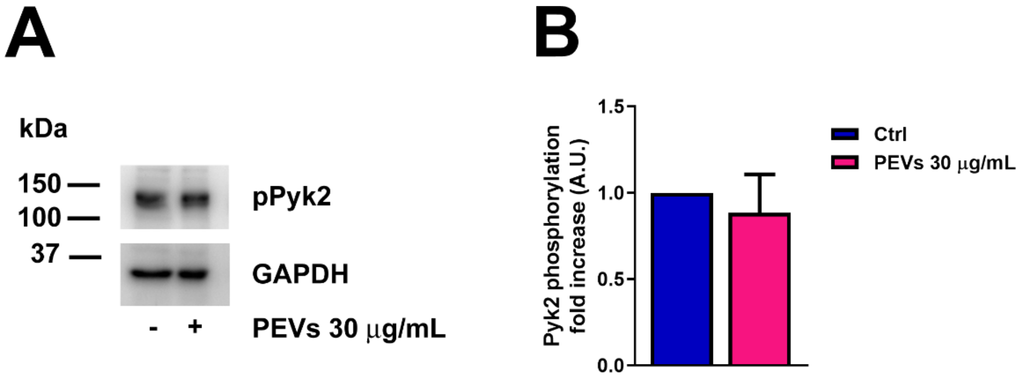

Figure S4. Long-term exposure of MDA-MB-231 cells with PEVs did not increase Pyk2 phosphorylation. Phosphorylation of Pyk2 **(A-B)** in MDA-MB-231 cells incubated with PEVs (30 μg/mL, 24 h) or left untreated. Representative immunoblots are reported in **A** for phospho-Pyk2 where GAPDH staining is for equal loading control. The quantification of the results is shown in **B** for phospho-pPyk2 as the mean ± SD of three experiments.

Table S1. Primers used for real-time qPCRs.

Target gene	Primer name	Primer sequence (5'–3')	Amplicon length (bp)
SERCA2B	SERCA2b_F1	ATGCCATTGTTCGAAGCCTC	136
	SERCA2b_R1	TCACCTTCCACTCTGTCCAG	
SERCA3	SERCA3_F1	TCCGCCTCATCGAGATCAAG	135
	SERCA3_R1	GCATGTTCTTCTTGTCCTGGT	
$InsP_3R1$	ITPR1_F	GGAGTTTCAGCCCTCAGTGG	101
	ITPR1_R	CTTCAGGCACAGAGACCAGG	
$InsP_3R2$	ITPR2_F	CACTCTGGGAAATAGAGGTGGTT	124
	ITPR2_R	TCAGGATTAAGCTCTGCAGCTA	
$InsP_3R3$	ITPR3_F1	GCTTCATCAGCACTTTGGGG	104
	ITPR3_R1	CTTGAAGAGGCAGTCACGGA	
STIM1	STIM1_F1	GCCCTGTGGCTCCTCTG	130
	STIM1_R1	TTCGGCAAAACTCTGCTGC	
Orai1	ORAI1_F	GCTTCGCCATGGTGGCAAT	127
	ORAI1_R	ATCATGAGCGCAAACAGGTG	
Actin beta[1]	ACTB_alt1_F	GGACCTGACTGACTACCTCAT	107
	ACTB_alt1_R	CGTAGCACAGCTTCTCCTTAAT	
Glyceraldehyde-3-phosphate dehydrogenase	GAPDH_alt_F	CGGGAAACTGTGGCGTGATGG	120
	GAPDH_alt_R	GCCATGCCAGTGAGCTTCCC	
